# Uncommon Presentation of Recurrent Lung Adenocarcinoma: A Finger Ulcer Induced by Subclavian Artery Invasion Successfully Healed With Viabahn VBX Treatment

**DOI:** 10.7759/cureus.55885

**Published:** 2024-03-10

**Authors:** Li Zhui, Jiang Chuli, Feng Yangyang, Zhao Yu, Ren Wei

**Affiliations:** 1 Department of Vascular Surgery, The First Affiliated Hospital of Chongqing Medical University, Chongqing, CHN

**Keywords:** covered stent, endovascular intervention, vascular invasion, recurrence, lung adenocarcinoma

## Abstract

Recurrence of a lung tumor invading the subclavian artery, causing stenosis and leading to finger ulcers as the initial symptom, is rare. We employed endovascular techniques, inserting a Viabahn® VBX covered stent (W. L. Gore & Associates, Flagstaff, Arizona) to aid in ulcer healing and improve the patient's quality of life. The patient, a 73-year-old male, had a history of lung adenocarcinoma resection two years prior but had not undergone follow-up examinations or cancer-specific treatments. Clinical examination revealed an invasion of the right subclavian artery by the recurrent tumor, resulting in severe stenosis and ischemic symptoms in the right upper limb. Given the patient's advanced cancer stage and the decline of further tumor-specific treatments, an endovascular intervention using a Viabahn VBX covered stent was performed to improve blood flow and promote ulcer healing. The stent demonstrated exceptional stability and patency during the six-month follow-up, greatly improving the patient's quality of life. This case highlights the importance of recognizing atypical symptoms as potential indicators of tumor recurrence or progression and demonstrates the promising role of covered stents in managing vascular complications in selected patients with advanced-stage malignancies.

## Introduction

Lung cancer, particularly adenocarcinoma, is the most common malignancy worldwide, with a five-year survival rate of around 19% [1]. Early identification of tumor recurrence is crucial for improving patient outcomes. Recurrent lung cancer typically presents with respiratory symptoms and invasion of nearby organs. The most common vascular invasion is that of the superior vena cava, leading to superior vena cava obstruction syndrome characterized by limb and facial swelling, as well as dilated veins in the neck and chest [2,3]. Conversely, clinical cases where tumor recurrence primarily presents with arterial invasion leading to limb ischemia are rare. The emergence of these atypical clinical features poses significant challenges in the early detection and subsequent management of malignant tumor recurrence in clinical practice.

Here, we report a case of a patient who underwent right upper lung adenocarcinoma resection two years ago and recently developed right upper limb weakness and persistent non-healing fingertip ulcers. Clinical examination revealed it was caused by the recurrence of right lung adenocarcinoma invading the right subclavian artery. We successfully performed endovascular intervention surgery by implanting a Viabahn® VBX covered stent (W. L. Gore & Associates, Flagstaff, Arizona), promoting ulcer healing, and greatly improving the quality of life for this advanced-stage cancer patient.

We present this case to encourage further research and discussions on optimal management strategies for patients with similar vascular complications stemming from recurrent malignancies, shedding light on new avenues for personalized and effective patient care.

## Case presentation

**Figure 1:**
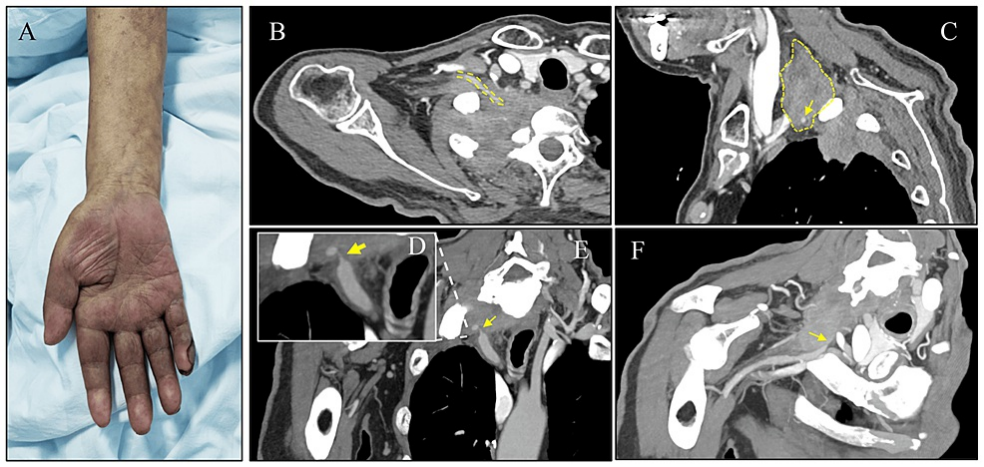
Radiological manifestations and clinical presentations of hand ischemia induced by right subclavian artery stenosis caused by malignant lung tumor. (A) Clinical signs of severe ischemia in the right upper limb: atrophy of the deltoid muscle and a right little finger ulcer. (B and C) Axial and sagittal views showing the malignant tumor encircling the right subclavian artery. (D and E) The proximal segment of the right subclavian artery stenosis observed in the coronal view. (F) The distal segment of the right subclavian artery stenosis observed in the coronal view. The yellow arrow indicates the lesion.

A 73-year-old male patient presented at the outpatient clinic with a chief complaint of weakness in the right upper limb for three months and ulceration on the right little finger for three weeks. The patient did not experience symptoms such as dizziness, headaches, or visual disturbances characteristic of subclavian steal syndrome. His medical history included a resection of lung adenocarcinoma two years prior, with no records of chemotherapy, and he had not undergone recent follow-up examinations. Physical examination revealed atrophy of the interosseous muscles in the right hand, a flattened palm, and a 5 mm×10 mm ulcer with gray-white purulent exudate on the outer side of the right little finger pulp (Figure 1A). The skin temperature of the right upper limb was significantly lower than that of the left, and the right radial artery was not palpable. Capillary refill time in the nail bed was prolonged. Muscle strength in the right upper limb was graded as 2, and the right brachial artery blood pressure was 65/34 mmHg, while the left brachial artery blood pressure was 128/77 mmHg. Blood test results were as follows: D-dimer, 10.2 mg/L; hemoglobin, 119 g/L; and fibrinogen, 5.4 g/L. Vascular ultrasound indicated severe stenosis at the beginning of the right subclavian artery. Enhanced chest CT revealed a mass in the apical segment of the right upper lobe, extending to the right thoracic inlet and paravertebral region. The mass invaded the right subclavian artery, causing significant stenosis at certain stages. The opening of the vertebral artery was not clearly displayed, and there were metastatic lesions in the seventh cervical and first thoracic vertebrae (Figure 1B and C). CTA angiography showed a linear filling defect inside the right subclavian artery, enveloped by the mass in the right upper lobe, indicating local invasion. The proximal diameter of the right subclavian artery was 8.7 mm, and the distal diameter was 4.1 mm (Figure 1D, E, and F).

**Figure 2:**
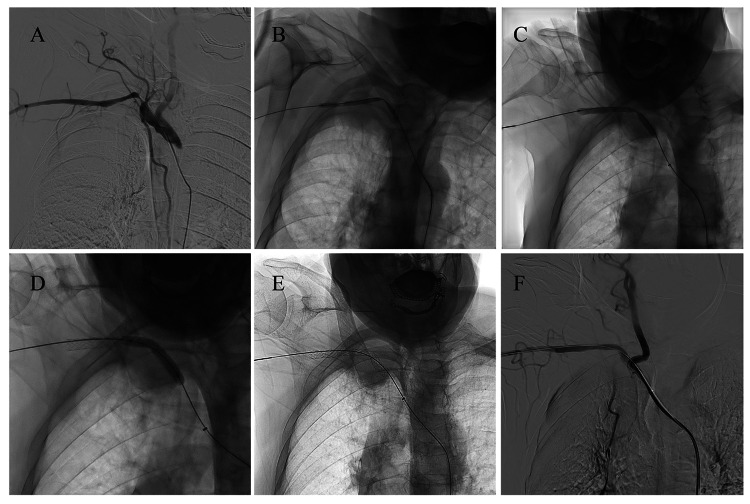
Endovascular procedure of covered stent implantation in the right subclavian artery. (A) Angiography confirms a lesion at the origin of the right subclavian artery, with the right vertebral artery not clearly visualized. (B) Balloon angioplasty (5 mm × 80 mm) predilates the lesion. (C) Viabahn VBX covered stent (7 mm × 79 mm) released using a balloon expansion technique. (D) Post-dilation (6 mm×40 mm balloon) ensures proper stent conformity in proximal and distal segments with the vessel wall. (E) The stent exhibits optimal conformation. (F) Improved blood flow to the upper limb observed after stent implantation.

**Figure 3:**
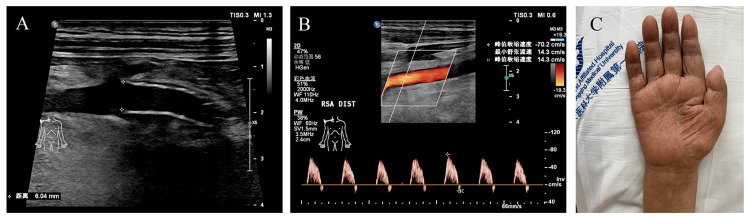
Six-month follow-up results. (A and B) Ultrasonography of the right subclavian artery demonstrates a well-formed intravascular stent (A) with unobstructed blood flow inside the stent (B). (C) Clinical manifestations of the right upper limb following endovascular treatment: erythematous skin color and complete healing of the ulcer on the right little finger.

Considering the recurrence of a malignant lung tumor with distant metastasis and the large size of the tumor, the patient was not eligible for tumor resection or open surgical vascular reconstruction. The patient declined further tumor-specific treatments but agreed to undergo intravascular reconstruction to improve symptoms of right upper limb ischemia. We performed the procedure through right femoral artery access. With the support of a stiff guidewire, a 90 cm 7F long sheath was advanced to provide support (Figure 2A). A 5 mm × 80 mm balloon (ZENFlow, Zylox Medical, Zhejiang, China) was used to dilate the target lesion, and then the long sheath was pushed to the distal segment of the lesion (Figure 2B). Subsequently, a Gore Viabahn VBX balloon-expandable covered stent (7 mm × 79 mm) was implanted. After withdrawing the long sheath, the stent was released and post-dilated using the stent's own balloon (Figure 2C) and a 6 mm × 40 mm balloon (Pacific™ Plus, Medtronic, Minneapolis, Minnesota) (Figure 2D) to ensure accurate deployment at the proximal and distal ends of the lesion. Post-procedure angiography confirmed significant improvement in distal blood flow, restoration of skin temperature in the right upper limb, and recovery of radial artery pulsation (Figure 2E and F). The right brachial artery blood pressure returned to 117/87 mmHg. After the procedure, the patient was prescribed long-term oral administration of clopidogrel 75 mg once daily. At 15 days post-operation, the ulcer on the right little finger had healed. On the six-month follow-up, ultrasound showed smooth blood flow within the subclavian artery stent, and both radial artery pulsation and brachial artery blood pressures were symmetrical on both sides (Figure 3). Unfortunately, it is with regret that the patient passed away from multiple organ failure during the seven-month follow-up.

Informed consent was obtained from the patient for the publication of this case report and any accompanying images.

## Discussion

We reported a rare case of recurrent lung adenocarcinoma with an unusual initial symptom of fingertip ulceration. The patient was successfully treated with a Viabahn VBX covered stent implantation to improve limb ischemia. Typically, lung malignancy recurrence presents with respiratory symptoms such as chest pain, tightness, dyspnea, cough, and hemoptysis. Distant metastasis may cause bone pain and headaches corresponding to the affected organs [4]. In cases where the initial clinical presentation of malignant tumors in the right upper lobe of the lung involves the vascular system, the most commonly affected vessel is the superior vena cava. This condition may lead to limb and facial swelling, jugular vein distension, chest wall venous collaterals, and acute manifestations such as laryngeal edema and elevated intracranial pressure [5]. However, in our case, tumor recurrence initially manifested as ischemic symptoms in the affected limb, progressively worsening over time and eventually resulting in a non-healing fingertip ulcer. This atypical symptom is relatively rare. Arteriosclerosis-related subclavian artery occlusion typically occurs at the proximal segment, leading to mild to moderate ischemic manifestations, such as limb weakness or decreased systolic blood pressure. Fingertip ulceration is seldom observed in these cases due to compensatory retrograde flow from the ipsilateral vertebral artery, known as the subclavian steal syndrome [6]. In our patient, the tumor affected the vasculature precisely at the opening of the right vertebral artery, and the patient had left vertebral artery dominance. As a result, there was no compensatory retrograde flow to supply blood flow to the right upper extremity, leading to severe ischemia and ulcer formation. Our case highlights the importance of recognizing unusual ischemic symptoms as potential indicators of tumor recurrence or progression, especially in patients with a history of lung tumors affecting nearby large blood vessels. Early detection can prompt timely intervention and improve patient outcomes. Therefore, clinicians should be vigilant in assessing atypical manifestations to impact treatment strategies and overall prognosis.

In this case, the patient's recurrent tumor has extensively invaded surrounding tissues and is accompanied by distant bone metastasis, making them unsuitable for tumor resection or open surgery for vascular reconstruction. Additionally, the patient has declined further chemotherapy, radiotherapy, and targeted therapy, thereby limiting the effectiveness of conventional cancer treatments. Given these challenges, alternative therapeutic strategies must be explored to alleviate the patient's symptoms and enhance their overall quality of life. Our team successfully implemented an endovascular reconstruction approach, involving balloon angioplasty and intravascular stent placement, to address the compromised blood flow in the affected limb. This intervention not only improved blood circulation but also promoted ulcer healing, restored the patient's muscle strength, and led to a significant enhancement in their quality of life, despite being in the advanced stages of cancer. As far as we know, this is the first reported case using a Viabahn VBX stent to improve ischemia caused by tumor invasion of the subclavian artery.

The right subclavian artery stenosis observed in this case presents distinct characteristics from arteriosclerosis. The primary cause of stenosis in this instance is the external compression and invasion of the blood vessel wall by the tumor. These factors cannot be adequately addressed solely through balloon angioplasty, as tumor invasion leads to elastic degeneration of the blood vessel wall, causing rapid recoil after balloon dilation. Therefore, intravascular stent placement is a necessary step to ensure the long-term patency and functionality of the artery [7].

Although the literature directly addressing stent selection for tumor-involved arterial lesions is limited, studies on patients with non-tumor-related subclavian artery occlusion have revealed, through a follow-up analysis of nearly 45 months, that the mid-term patency rate of covered stents was significantly higher than that of bare stents [8]. Additionally, research on malignant superior vena cava obstruction demonstrated the superiority of covered stents over bare-metal stents in terms of long-term patency rates [9]. A randomized controlled study conducted by Gwon et al. showed a significant decline in the patency rate of bare-metal stents at the third month after treatment (covered stent vs. bare-metal stent: 94% vs. 79%). In contrast, the patency rate of covered stents remained consistently above 94% at 12 months, while bare-metal stents exhibited a substantially lower patency rate of only 48% [10]. Furthermore, considering that without chemotherapy or targeted therapy to suppress tumor growth, the tumor's progression may lead to rapid re-occlusion of bare-metal stents. Therefore, covered stent implantation may offer a more promising solution for patients with an expected lifespan exceeding three months.

In this case, we opted for the Viabahn VBX covered stent due to its exceptional radial support, flexibility, and adaptability, making navigation and deployment easier. Its ability to post-dilate the device diameter by up to +4 mm is particularly suitable for our case, where there was nearly a 5 mm difference between the proximal and distal anchoring zones. This unique design allowed the stent diameter to adjust effectively with balloon dilation, making it highly appropriate for the patient's condition. Furthermore, the stent's coating with CARMEDA BioActive Heparin, derived from pigs and characterized by stable, covalently bound low molecular weight heparin, ensures excellent biocompatibility and vascular surface characteristics. This choice was particularly advantageous, given the patient's malignant tumor and hypercoagulable state, as it effectively reduced the risk of thrombosis after stent placement. In conclusion, the unique design of the Viabahn VBX covered stent provides a reliable solution for maintaining long-term patency in treating limb ischemia caused by tumor invasion of the blood vessel, making it well-suited for our patient's specific condition.

During the six-month follow-up period, the stent demonstrated remarkable stability, with no signs of displacement or fracture observed. In comparison, Perri et al. reported instances of stent folding and thrombosis at one and four months during the treatment of pseudoaneurysms of the subclavian artery resulting from clavicle fractures. Nonetheless, those complications were successfully addressed through balloon angioplasty and clavicle plate adjustment, leading to the restoration of normal stent shape and patency [11]. These complications were likely attributed to external compression and limb movement, contributing to stent occlusion. Remarkably, our patient did not encounter similar complications despite external tumor compression. However, it is essential to acknowledge the limitations of this case report. Firstly, as a single case study, the findings may not be generalizable to all patients with similar conditions. More extensive studies and clinical trials are necessary to validate the efficacy and safety of endovascular interventions using covered stents in cases of tumor-involved arterial lesions. Secondly, the long-term follow-up of this patient is limited to six months. Further investigations with extended follow-up periods will provide a more comprehensive understanding of the stent's durability and patency in this context.

## Conclusions

This case highlights the atypical clinical presentation of tumor recurrence with vascular invasion in lung adenocarcinoma, leading to ischemic symptoms in the upper limb. Endovascular reconstruction with the Viabahn VBX covered stent proved to be a safe and effective approach in alleviating the patient's symptoms and improving their quality of life. The use of covered stents in such cases is promising, providing long-term patency in compromised vessels due to tumor invasion. Further research and prospective studies are warranted to establish the optimal management strategies for patients with similar vascular complications resulting from malignancies. The success of this case underscores the importance of considering endovascular interventions in carefully selected patients, particularly when traditional surgical resection is not feasible or declined by the patient.
